# Comparison of Ultrasonic and CO_2_ Laser Pretreatment Methods on Enzyme Digestibility of Corn Stover

**DOI:** 10.3390/ijms13044141

**Published:** 2012-03-29

**Authors:** Shuang-Qi Tian, Zhen-Yu Wang, Zi-Luan Fan, Li-Li Zuo

**Affiliations:** 1 School of Food Science and Engineering, Harbin Institute of Technology, Harbin 150090, China; E-Mails: tianshuangqi2002@163.com (S.-Q.T.); fzl_1122@163.com (Z.-L.F.); zuolili213@163.com (L.-L.Z.); 2 College of Food science and Technology, Henan University of Technology, Zhengzhou 450001, China; 3 School of Forestry, Northeast Forestry University, Harbin 150040, China

**Keywords:** pretreatment, CO_2_ laser, ultrasonic, corn stover, FT-IR

## Abstract

To decrease the cost of bioethanol production, biomass recalcitrance needs to be overcome so that the conversion of biomass to bioethanol becomes more efficient. CO_2_ laser irradiation can disrupt the lignocellulosic physical structure and reduce the average size of fiber. Analyses with Fourier transform infrared spectroscopy, specific surface area, and the microstructure of corn stover were used to elucidate the enhancement mechanism of the pretreatment process by CO_2_ laser irradiation. The present work demonstrated that the CO_2_ laser had potential to enhance the bioconversion efficiency of lignocellulosic waste to renewable bioethanol. The saccharification rate of the CO_2_ laser pretreatment was significantly higher than ultrasonic pretreatment, and reached 27.75% which was 1.34-fold of that of ultrasonic pretreatment. The results showed the impact of CO_2_ laser pretreatment on corn stover to be more effective than ultrasonic pretreatment.

## 1. Introduction

The new bio-refinery industries benefit the nation’s energy security, economic progress, and environmental protection in many different ways [1,2]. However, current data suggests that only bioethanol offers large scale reductions in greenhouse gas emissions compared with non-renewable fossil fuels. As a promising alternative fuel today, bioethanol is mostly obtained from sugar or starchy materials that are tolerably expensive. However, lignocellulosic biomass may be utilized to produce bioethanol, which is a promising alternative energy source for non-renewable crude oil [3]. Corn stover is one of the greatest potential annual crop-based bioethanol feedstocks, because it is made up of three components, lignin, cellulose, and hemicellulose. Of these both cellulose and hemicellulose can be hydrolyzed to fermentable monomeric sugars, which can then be converted to bioethanol using pentose and hexose fermenting substance [4]. The so called cellulosic bioethanol in the long run shows more value than grain bioethanol. Exploring renewable resources for bioethanol production has been underway for many years [5]. However, this necessitates a pretreatment process to break up the highly ordered structure of the lignocellulosic biomass and to remove the lignin materials so as to expose the hemicellulose and cellulose parts to the related enzymatic action [6]. The effect of physical pretreatment falls short of expectations, and chemical pretreatment produces environmental pollution and requires corrosion-resistant, high quality reactors. Bioethanol from non-grain biomass has been a dream encumbered by technological and economic factors [7].

Pretreatment processes, which can disrupt cellulose crystallinity and increase the porosity of the biomass, have been shown to be effective in enhancing the hydrolysis process of corn stover. Several studies have been exploited, such as ultrasonic, microwave, AFEX, ARP, alkaline pretreatment, dilute sulfuric acid, and liquid hot water [8]. The effectiveness of a physical method is insufficient for an optimum performance, whereas chemical pretreatment methods are still encountering many problems such as cost-effectiveness, high-power usage, environmental contamination etc. [9]. Little research work has been performed on the pretreatment of lignocellulose with lasers, and ultrasonic pretreatment is the most conventional pretreatment method. In this study, we compared the effect of ultrasonic and CO_2_ laser pretreatment methods on the enzyme digestibility of corn stover.

CO_2_ lasers are the highest-power continuous wave lasers that are currently available [10]. As a novel method, the pretreatment on corn stover with a CO_2_ laser was developed, and the difference between ultrasonic and CO_2_ laser pretreatment on the enzyme digestibility of corn stover analyzed.

## 2. Results and Discussion

### 2.1. Compositional Analysis of Raw Corn Stover Sample

Analyses according to the modified Van Soest analytical procedures were performed to the composition (%, w/w, dry weight basis) in raw corn stover materials [11]. The results showed that the raw sample contained 36.7 ± 1.13% cellulose, 35.5 ± 1.51% hemicellulose, 5.3 ± 0.47% lignin, 1.9 ± 0.11% ash, and 4.1 ± 0.13% moisture. The presence of 72.2% holocellulose makes stover a potential and renewable biomass source for bioethanol production [12].

### 2.2. Enzymatic Hydrolysis

**Figure 1:**
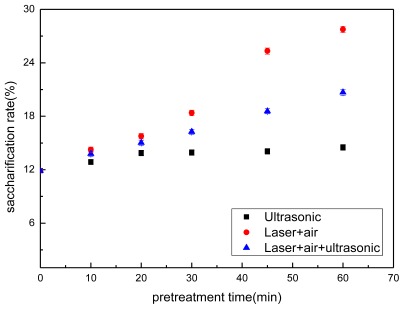
Effect of enzymatic hydrolysis with different pretreatments.

Corn stover biomass represents a readily available recyclable feedstock which can be used in the production of bioethanol and a variety of chemicals. Lignocellulosic biomass cannot be hydrolyzed by cellulase at a high rate of recovery without a rational pretreatment procedure because the lignin in the biomass is a barrier to cellulase action. A report indicated that pretreatment of corn stover enhanced the saccharification efficiency of lignocellulosic biomass [13]. As shown in Figure 1, the results also show that, compared with that of non-pretreatment (without being subject to pretreatment), all kinds of pretreatments, including LAM, UP, and LAUP, increase the enzymatic conversion rate. Error bars indicated one standard deviation (three replicate experiments). As for the non-pretreated corn stover, the saccharification efficiency only reached a level of 11.89%. The saccharification efficiency of pretreated stover was increased in a nonlinear fashion with increasing time. LAM showed that the saccharification efficiency was significantly higher than UP and LAUP pretreatments, and reached 27.75% after 48 h hydrolysis. These results suggested that the efficiency of LAM was higher than the other two pretreatments, and was the most favorable for cellulase enzymatic hydrolysis. LAM and UP had the most different pretreatment methods and mechanisms, therefore LAM and UP were determined with regard to sugar generation and FTIR analysis.

### 2.3. Effect of Pretreatment on Sugar Content Hydrolysate

HPLC analytic results showed that glucose, xylose and cellobiose were the major components in the final enzymatic hydrolysates for all pretreated samples (Table 1). However, non-pretreated corn stover was an exception, the cellobiose was almost not detectable in the hydrolysates. In the LAM hydrolysate, the concentrations of xylose, glucose and cellobiose were 15.03, 131.20 and 4.67 mg/g corn stover, respectively, while there were 20.61, 101.13 and 7.07 mg/g corn stover in the UP hydrolysate, respectively, and in non-pretreatment hydrolysate, xylose and glucose concentrations were 5.33, 15.00 mg/g corn stover, and a little cellobiose. In the LAM hydrolysate, only a small amount of xylose was detected, and the ratio of xylose to glucose in the liquid was about 1:9. However, the concentration of xylose in the UP hydrolysate achieved a high concentration of 20.61 mg/g corn stover, and the ratio was about 1:2.5. The cellobiose was almost undetected in the non-pretreatment hydrolysate, but lots of impurities emerged in the hydrolysate. The results showed that LAM pretreatment could remove lignin and part of hemicellulose, and hence, improve the hydrolysis of cellulose significantly. Therefore, the effectiveness of LAM pretreated corn stover is remarkably better than UP pretreated corn stover.

### 2.4. Analysis of the FT-IR Spectroscopy

**Figure 2:**
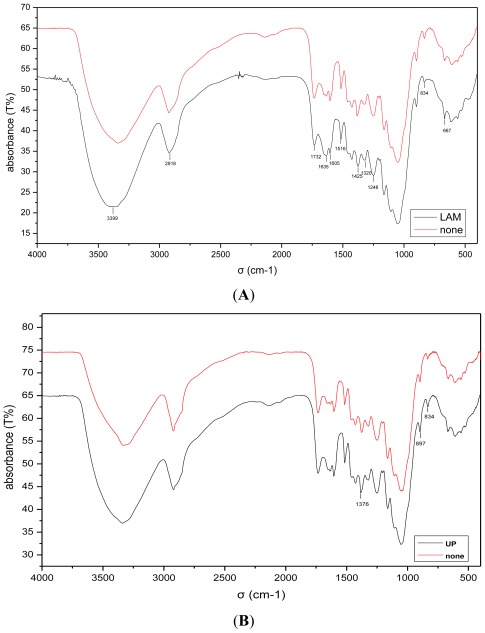
FT-IR Spectra of the different pretreatment of corn stover. (**A**) LAM pretreated corn stover sample compared with non-pretreated; (**B**) UP pretreated corn stover sample compared with non-pretreated).

Analysis with FT-IR spectroscopy was used to elucidate the enhancement mechanism of the hydrolysis process. FT-IR was calibrated and considered as a valid means of analyzing the properties of chemical bonds in the biomass after the pretreatment process (Table 2) [14]. The FTIR spectra of non-pretreated, LAM and UP pretreated samples depicted that there were many differences present in the master region (1735–650 cm^−1^) which correspond to lignin infrared fingerprints. As shown in Figure 2A, compared to the non-pretreated samples, the LAM pretreated substrate showed a reduction in the bands at 1732 and 1248 cm^−1^, which is indication of C=O stretching and hemicellulose-lignin linkage due to carbohydrate linked with lignin, respectively. At 2918 cm^−1^, the –CH_2_- and –CH_3_ asymmetric and symmetric scissoring deformations can be observed. The aromatic skeletal vibrations are assigned at 1605 and 1422 cm^−1^. The FT-IR spectra showed that some degradation and solubilization of carbohydrate and lignin occurred in pretreated corn stover samples. However, an increase in the intensity of peaks at 1635, 1320, 1516, and 667 cm^−1^ (aromatic ring stretch) in the pretreated corn stover compared to the non-pretreated sample was noticed in the spectra [15]. The intensity of these peaks was stronger in the pretreated corn stover compared with non-pretreated corn stover indicating an increase in surface lignin after LAM pretreatment. The increase in the surface lignin concentration in pretreated corn stover compared to non-pretreated materials was attributed to release of lignin on the lignocellulose surface. While aromatic C–H out of bending exhibits at 837 cm^−1^, the results also showed release of the lignin in the lignocellulose biomass. However, in the UP pretreated corn stover, only a little bonds change was found (Figure 2B).

### 2.5. Effect of Pretreatments on Morphology

**Figure 3:**
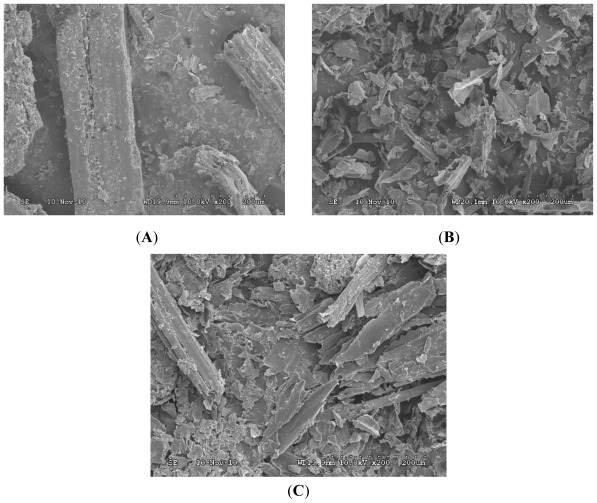
Scanning electron microscope samples of corn stover surface features: (**A**) non-pretreatment 200×; (**B**) LAM pretreatment 200×; (**C**) UP pretreatment 200×.

The morphology of the non-pretreated and pretreated corn stover with different methods was investigated by scanning electron microscopy (SEM) [16]. SEM images showed that lignocellulose in the non-pretreated corn stover had an intact surface structure (Figure 3A), while LAM pretreatment disrupted the lignocellulosic structure mainly by breaking up corn stover lignin. As a result, the structure of major microfibrous cellulose was retained (Figure 3B) and some lignin or lignin-carbohydrate complexes were condensed on the surface of the biomass. At the same time, the contiguous, smooth surface of non-pretreated corn stover was perforated by the LAM pretreatment processes. These polyporous structures increased the cellulase-accessible surface area which increased the cellulase digestibility of the pretreated corn stover, and by feeling the material with the hand, the results showed that LAM biomass was much softer than the non-pretreated corn stover. Therefore, it positively increases the external surface region and the porosity of the biomaterials. However, the effect of the LAM pretreatment processes was very different from the UP pretreatment processes. As shown in Figure 3C, the destruction and fragmentation degree of corn stover in the LAM pretreatment processes was more significant than with the UP pretreated corn stover samples.

### 2.6. Mechanisms of LAM Pretreatment

**Figure 4:**
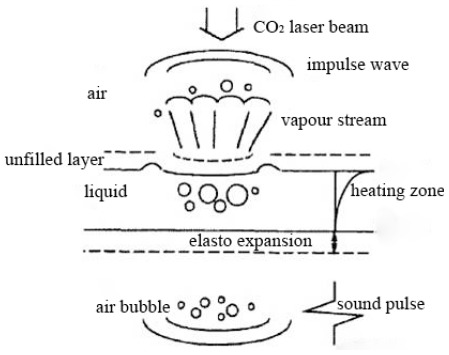
Laser in a liquid medium incentive mechanism model.

It has been reported that CO_2_ laser pretreatment could improve the enzymatic hydrolysis of corn stover for production of fermentable monomeric sugars [17]. However, researchers have had limited knowledge about CO_2_ laser and its effects. We speculated that the mechanisms of CO_2_ laser pretreatment might include three major contributions (Figure 4): (1) when the laser pulse energy density becomes smaller, liquid, caused by the nonlinear heating temperature gradient, induces thermal expansion and thermoelastic stress that emanates from the endothermic area and spreads the sound waves; (2) when the laser pulse energy density becomes greater, the temperature of the liquid is heated above the boiling point which leads to vaporization, evaporation and boiling of liquid caused by the expansion of steam burst; (3) when using higher laser energy density irradiates on the surface of a liquid, the surface or the depth of the liquid will produce laser plasma. After which, the plasma continues to absorb the laser energy, and ultimately leads to “an explosion” that results in optical breakdown of water, thus generating shock waves in the water [18,19]. It has been reported that microwave irradiation is one of the effective methods that has been explored for pretreatment of lignocellulosic materials [20–23] because of its high and selective energy transfer efficiency. Other researchers have used ultrasonic at higher energy levels to degrade lignocellulose which resulted in a reduction in viscosity [24,25].

## 3. Experimental Section

### 3.1. Materials and Equipment

**Figure 5:**
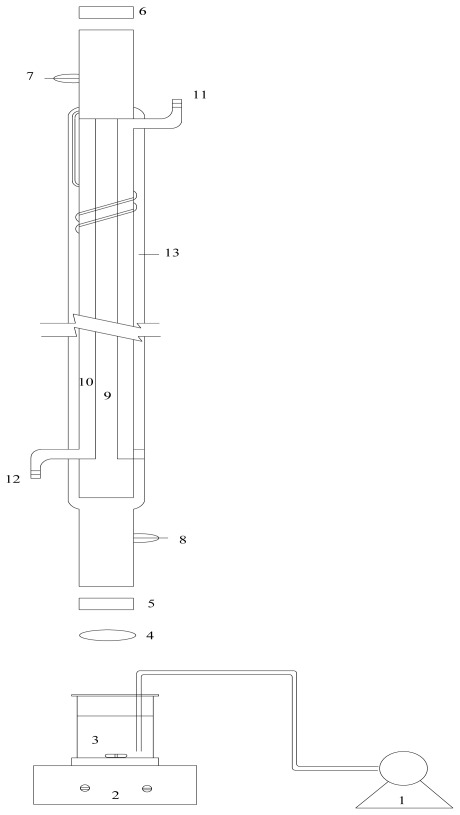
Mode chart of CO_2_ laser pretreatment of corn stover. 1: air pump; 2: magnetic stirrer; 3:bioreactor; 4: laser optical lens; 5: output reflector; 6: input reflector; 7: electrode (+); 8: electrode (−); 9: discharge tube; 10: condensing tube; 11: water outlet; 12: water inlet; 13: air container.

Corn stover was grown in a rural region near Harbin city, China, and harvested in the fall of 2010. The biomass was dried in air, chopped into short pieces with lengths ranging from 1 to 2 mm by a chipper mill (Tianjin Taisite Instrument Co., Ltd., Tianjin, China). Afterwards, the treated corn stover chips were kept in a refrigerator (4 °C) before using. Crude cellulase powder was purchased from Gansu Hualing Biological Technology Co., Ltd. Cellulase activity was determined by filter paper activity (FPA), and the activity was 74.88 FPU/g cellulase loading [26]. The apparatus used for the pretreatment reactions was a CO_2_ laser equipment designed by HIT, which is made up of two parts, CO_2_ laser tubes (0–300 W) and some other accessories, such as air pump, magnetic stirring apparatus, and reaction center (Figure 5) [17]. The CO_2_ laser tube was purchased from Nanjing Latron Laser Technology Co., Ltd.

### 3.2. Composition of Corn Stover Biomass

The untreated corn stover was dried in an oven at 60 °C to constant weight, and the composition of corn stover analyzed. Cellulose, hemicellulose, lignin, ash, and moisture were analyzed following the modified Van Soest forage analytical procedures.

### 3.3. Pretreatment of Corn Stover Samples

The pretreatment of corn stover was performed by three different methods: (1) CO_2_ laser at 150 W which was carried out with air pretreated corn stover for 0–60 min attended by magnetic stirring (LAM); (2) The frequency at 150 W of ultrasonic pretreatment (UP) was used for 0–60 min on corn stover; (3) LAM was combined with ultrasonic pretreatment (LAUP) at 150 W for 0–60 min. The condition of pretreatment hydrolysis was carried out at 5.0% (w/v) solid content for time periods (10, 20, 30, 45 and 60 min). After the pretreatment, the bioreactor was immediately cooled down in a water bath to maintain room temperature. The pretreated products were filtered and washed with distilled water. After pretreatment, the samples were hydrolyzed by crude cellulase powder.

### 3.4. Enzymatic Hydrolysis of Pretreated Corn Stover

The pretreated corn stover was hydrolyzed for 48 h at 45 °C and pH 5.5 and S/L ratio of 2% (w/v) in a shaking bath (160 rpm). Enzyme concentrations of 179.7 FPU/L were applied to all the samples, including non-pretreated, LAM, UP and LAUP. For all the samples, the sample solution was taken out, and centrifuged at 12,000 g and 4 °C for 6 min. The obtained supernatant was kept at 80 °C for 10 min and then used for total reducing sugars assay with the DNS method. The formula below was used to calculate the enzymatic hydrolysis of corn stover saccharification efficiency [27]. Standard deviation (based on three replicates) found in different corn stover samples (without and with pretreatment).

(1) $$\eta =\frac{R_{s}\times 0.9}{(\omega c+\omega hc)\times m}\times 100\%$$

where *η* is the saccharification rate (%); *m* is the hydrolytic sample content, mg; *R_s_* is the total reducing sugars (mg/mL); *ωc* is the mass fraction of cellulose and *ωhc* is the mass fraction of hemicellulose in the corn stover samples, respectively.

### 3.5. Hydrolysate Analysis

The monomeric sugars after enzymatic hydrolysis were measured using a HPLC (Agilent Technologies Co., Ltd., CA, USA) with a Shodex sugar column SP0810 at 35 °C with acetonitrile: water (3:1) at 1 mL/min as the mobile phase. The HPLC system was equipped with a refractive index detector which quantified the monomeric sugars.

### 3.6. Spectral Analysis of Pretreated Samples

The pretreated samples were extruded uniformly against the diamond surface with a spring-loaded anvil, and FT-IR spectra were acquired by averaging 16 scans from 4000 to 400 cm^−1^ at resolution 1 cm^−1^. ATR corrections were applied by Spectrum One software supplied with the device.

### 3.7. Surface Morphology Observation by SEM

The samples for SEM analysis were taken from corn stover with different pretreatments, as stated previously. The samples were sputter-coated with Au-Pd prior to imaging with Scanning Electron Microscope (JEOL 100 CX II-ASID 4D, Tokyo, Japan) using an accelerating voltage of 10 kV. The SEM was equipped with an energy-dispersive X-ray (EDX) analyzer, which offered useful high definition images.

### 3.8. Statistical Analysis

All data were estimated in triplicate, and the data were expressed as average values. All saccharification efficiency data were standardized by expressing the pretreated corn stover formation and reducing sugar for the samples as the pretreatment effect of the biomass and reducing sugar relative to the control without pretreatment. Statistical calculations were carried out using the statistical analysis software Origin (version 8.5; OriginLab: Northampton, MA, USA, 2011).

## 4. Conclusions

It is believed that CO_2_ laser pretreatment, unlike chemical or thermal decomposition, is a non-random process with irradiation taking place specifically at the centre of the corn stover aqueous solution system and with larger molecules degrading the fastest [28,29]. The study showed that the CO_2_ laser pretreatment could improve the release of the cellulose in the pretreated biomass and the efficiency of lignocellulose breakdown. Currently, the cost-performance and running costs of CO_2_ laser pretreatment are expensive, because large amounts of electricity are required. However, Takashi Yabe has found that solar power could transform lasers, which thus favors laser pretreatment [30]. The influence on the sugar content hydrolysate of LAM pretreatment on corn stover is more significant than UP. The LAM laser pretreatment is a novel process to treat corn stover, and has great potential for pretreatment of lignocellulosic materials in the bioethanol industry.

## Figures and Tables

**Table 1:** Major reducing sugar component contents in different pretreatment hydrolyzates.

Sample	Xylose (mg/g biomass)	Glucose (mg/g biomass)	Cellobiose (mg/g biomass)
CO_2_ laser pretreated hydrolysate	15.03	131.20	4.67
Ultrasonic pretreated hydrolysate	20.61	101.13	7.07
Non-pretreatment hydrolysate	5.33	15.00	trace amount

**Table 2:** FTIR absorption peak location and assignment of corn stover.

Wave Number(σ/cm^−1^)	Intensity of Absorption Band	Absorption Peak Assignment
3338	steep	OH stretching in alcohol and phenol
2921	moderate	C–H symmetrical and asymmetrical stretching in –CH_3_ and –CH_2_– Organic acid COO– asymmetrical stretch
1650–1630	semi-steep	Lignin and aromatic ring conjugated C=O stretch
1509–1515	moderate	Lignin and other aromatic ring skeletal stretch –CH_2_– scissoring deformation in carbonhydrates and fatty compounds
1462	infirm	C–H deformations (asym. in –CH_3_ and –CH_2_–) in lignin and carbonhydrates
1421	steep	Aromatic skeletal vibrations combined with C–H in-plane deformations
1325	moderate	C–H vibration in cellulose and C_1_–O vibration in syringyl derivatives
1265	moderate	Aromatic skeletal vibrations, guaiacyl, C=O stretch
1160	faint	C–O–C vibration in cellulose and hemicellulose
1117–1124	infirm	C–H aromatic ring, syringyl C–O stretch in cellulose and hemicellulose
1049	moderate	Si–O stretch in amorphous SiO_2_
898	faint	C–H deformation in cellulose and saccharide
666	faint	Single-plane vibration of substituted aromatics
